# Malnutrition among the Elderly in Malaysia and Its Associated Factors: Findings from the National Health and Morbidity Survey 2018

**DOI:** 10.1155/2021/6639935

**Published:** 2021-04-12

**Authors:** Mohamad Hasnan Ahmad, Ruhaya Salleh, Cheong Siew Man, Munawara Pardi, Norsyamlina Che Abdul Rahim, Norhafizah Shahril, Mohd Hatta Abdul Mutalib, Suzana Shahar, Noor Ani Ahmad

**Affiliations:** ^1^Institute for Public Health, National Institutes of Health, Ministry of Health Malaysia, 41070 Shah Alam, Selangor, Malaysia; ^2^Centre for Healthy Aging and Wellness, Faculty of Health Sciences, Universiti Kebangsaan Malaysia, 50300 Kuala Lumpur, Malaysia

## Abstract

**Background:**

Malaysia is predicted to become an ageing population by 2035. Malnutrition among the elderly is one of growing concern. This study aims to identify the prevalence of malnutrition and its associated factors among the elderly in Malaysia.

**Methods:**

Data from the National Health and Morbidity Survey (NHMS) 2018 was analysed. This survey applied a multistage stratified cluster sampling design to ensure national representativeness. Malnutrition was identified using a validated Mini Nutrition Assessment-Short Form (MNA-SF). Variables on sociodemographic, health status, and dietary practices were also obtained. The complex sampling analysis was used to determine the prevalence and associated factors of at-risk or malnutrition among the elderly.

**Result:**

A total of 3,977 elderly completed the MNA-SF. The prevalence of malnutrition and at-risk of malnutrition was 7.3% and 23.5%, respectively. Complex sample multiple logistic regression found that the elderly who lived in a rural area, with no formal or primary level of education, had depression, Instrumental Activity of Daily Living (IADL) dependency, and low quality of life (QoL), were underweight, and had food insecurity and inadequate plain water intake were at a significant risk of malnutrition (malnutrition and at-risk), while Chinese, Bumiputra Sarawak, and BMI more than 25 kgm^−2^ were found to be protective.

**Conclusions:**

Currently, three out of ten elderly in Malaysia were at-risk or malnutrition. The elderly in a rural area, low education level, depression, IADL dependency, low QoL, underweight, food insecurity, and inadequate plain water intake were at risk of malnutrition in Malaysia. The multiagency approach is needed to tackle the issue of malnutrition among the elderly by considering all predictors identified from this study.

## 1. Introduction

A longer life brings many opportunities. However, it probably brings some adverse impacts if we do not correctly manage their life, especially on their health care. [1] The World Health Organization (WHO) estimated, between 2015 and 2050, the proportion of the world's population over 60 years old will nearly double from 12% to 22% and by the year 2020, the number of people aged 60 years and older will outnumber children younger than five years. [2] Every country is experiencing growth in the size and proportion of the elderly in their population. [3] Therefore, all nations will face significant challenges on how to ensure that their health care and social systems are ready to support the ageing population [2, 3].

Malnutrition among the elderly is one of the most growing concerns on this demographic shift. During the change into older years, often nutrition priorities change towards meeting and minimizing increased nutrient needs with fewer energy requirements and also preventing lean muscle loss [4]. Malnutrition in the elderly leads to protein-energy malnutrition, sarcopenia and cachexia. The protein-energy malnutrition increases with age and the number of comorbidities [5].

Malnutrition among the elderly is related to multifactorial causality [6]. It starts with factors affecting food consumption such as financial constraints, empty nest syndrome, a decrease of sensory function, oral health problems, gastrointestinal problems, polypharmacy, and many others. Then, these factors link to the difficulty of obtaining food, poor appetite and impaired ingestion, digestion, and absorption that will manifest malnutrition among the elderly [5, 6]. Because of the impact on the elderly, malnutrition entered the arena of the “geriatric giants” and is standing side by side with traditional geriatric syndromes like immobility, instability, incontinence, and intellectual impairment. [6, 7].

Malaysia is predicted to become an ageing population by 2035 [8]. Interestingly, despite the social responsibilities and challenges encountered and the policies implemented by the government, there is still much more the government needs to do to overcome the challenges faced by the ageing society in Malaysia [9]. Continuous effort is necessary to prepare a better health care delivery model and a more sustainable health system for preventing malnutrition among the elderly in this country [10]. A comprehensive specific country data on malnutrition among the elderly is needed to ensure this to materialize. Therefore, this study aims to identify the prevalence of malnutrition and its associated factor among the elderly in Malaysia.

## 2. Materials and Methods

### 2.1. Study Design

This study is part of a community-based nation-wide cross-sectional survey conducted to assess the health status of the elderly in Malaysia. This survey applied a multistage stratified random sampling design to ensure the selection of a nationally representative sample. Details of the methodology of this survey are explained in the published technical report [11].

### 2.2. Sample Size Estimation

The sample size was calculated using a single proportion formula for estimation of prevalence with adjustments for the total number of target population, design effect, expected nonresponse rates of 30% and to the need of the analysis by stratification of urban and rural. Based on the estimated prevalence of malnutrition or at-risk of malnutrition among the elderly of 42.5% from a local study, a total of 3,977 elderly were enough for the analysis [12].

### 2.3. Study Population

All the elderly aged 60 years and above from the randomly selected living quarters were invited to join this study. Those who were blind or deaf, illiterate, or found as having probable dementia, scored ten or below using Identification and Intervention for Dementia in Elderly Africans (IDEA), were excluded. Research ethical approval was obtained from the Medical Research & Ethics Committee's (MREC), Ministry of Health Malaysia (KKM/NIHSEC/P18-49(6)) on 18 January 2018 before starting the data collection.

### 2.4. Tools and Instrument

Malnutrition status was assessed using a validated Malay version of the Mini Nutrition Assessment-Short Form (MNA-SF). [13] The MNA-SF test comprises six questions that can be completed in few minutes: anthropometric measurements (body mass index, weight loss); global assessment (mobility); and dietary questionnaire and subjective assessment (food intake, neuropsychological problems, acute disease). The total scores of MNA-SF are ranging from 0 to 14. The range of overall score below 8, 8 to11, and above 11 indicates malnutrition, risk of malnutrition, and no malnutrition or normal, respectively. [14].

Other information also being collected from eight modules is as follows: (1) sociodemographic characteristics; age, sex, marital status, highest education levels, individual monthly income, and living arrangement; (2) self-reported or current medical report assessment on noncommunicable diseases status which were diabetes, hypertension, and hyperlipidaemia; (3) functional limitations which include Activities of Daily Living (ADL) using 10-item Barthel's ADL to assess personal self-care in getting ready for the day [15] and Instrumental Activities of Daily Living (IADL) by the Lawton's IADL scale to assess more complex sets of skills we need in order to live independently [16]; (4) social support evaluated based on the Duke's Social Support Index [17]; (5) dietary practice which includes fruit, vegetable, and water intake per day; (6) food security using 6-item Short Form of Food Security Status by the United States Department of Agriculture [18]; (7) quality of life using the Control, Autonomy, Self-Realization and Pleasure (CASP-19) scale [19]; and (8) depression using Geriatric Depression Scale (GDS) [20].

### 2.5. Respondent Recruitment and Data Management

Listing activities to identify eligible respondents (elderly aged 60 years and above) from the selected living quarters was conducted before the data collection. The face-to-face interviews were carried out by trained research assistants using mobile devices with structured questionnaires in tablet application. The application has an in-built quality control to ensure valid responses, with real-time data entry to our server at the institute. Self-administered modules which were filled up by the elderly in scannable paper format were sealed into envelopes and mailed to our institute for data processing.

### 2.6. Data Analysis

Data were analysed using complex samples module using the IBM Statistical Package for the Social Sciences (SPSS) software for Windows, Version 26.0. The prevalence of malnutrition and risk of malnutrition were calculated using complex samples frequencies. For logistic regression analysis, the data of at-risk and malnutrition were combined into the same group named malnutrition. The associations between malnutrition and other variables were evaluated using complex sample logistic regression, with 95% confidence intervals, significance level at the *p* value of 0.05, and control for all possible confounding factors.

## 3. Results

A total of 3,977 respondents aged 60 years and above were successfully recruited and completed MNA-SF. The distribution was almost equal by sex, and the majority of them stayed in urban areas. The distribution of respondents by age group and ethnicity mirrored the Malaysian population characteristic, as shown in Table 1.

Overall, the prevalence of malnutrition and at-risk of malnutrition by MNA-SF among the elderly in Malaysia was 7.3% and 23.5%, respectively. The prevalence of malnutrition and at-risk of malnutrition was higher in rural areas, among the older age group, among Indian, bumiputra Sabah, and other ethnicities, among unmarried or separated or divorced elderly, among lower education, among unemployed, and among lower individual income as shown in Table 2.

Complex sample multiple logistic regression among 3472 respondents found that the elderly who lived in rural areas, with no formal or primary level of education, had depression, IADL dependency, and low quality of life, were underweight, and had food insecurity and inadequate plain water intake were significantly associated with at-risk or malnutrition. Meanwhile, elderly Chinese, elderly Bumiputra Sarawak, and the elderly with BMI more than 25 kgm^−2^ were found to be significantly associated with at-risk or malnutrition with the odds ratio less than one as shown in Table 3.

## 4. Discussion

Prevalence of malnutrition and at-risk of malnutrition among the elderly in Malaysia was 7.3% and 23.5%, respectively. This finding was comparable with a neighbouring country data where a cross-sectional study of a population-based cohort in Singapore reported that the prevalence of malnutrition was 2.8%, and at risk of malnutrition was 27.6% [21]. Meanwhile, a study conducted in Sri Lanka found that 52.4% of the elderly were at risk of malnutrition, and 12.5% were in the malnourished range of MNA scores [22]. The prevalence of malnutrition in Singapore was lower than in Malaysia and Sri Lanka, probably because Singapore is a developed country all area is urban, and it had better health service coverage [23].

However, the prevalence reported in this national survey was lower compared to finding from several local smaller-scale studies. A study among Malay older adults in Felda Sungai Tengi, Selangor, in 2013 showed that 42.5% of the elderly were malnourished or at risk of malnutrition [12]. Another local study conducted in Kuantan, Pahang, in 2016 found that 64% of the elderly were at risk of malnutrition [24]. The disparity in these prevalence is due to the fact that these local, small-scale studies focused on a particular region and target community, such as rural areas and older people who are more vulnerable to the risk of malnutrition, as opposed to the NHMS 2018, which took samples to reflect the entire elderly population in Malaysia [25]. It also is proven in this survey that the prevalence of at-risk or malnutrition was higher in rural areas, among older age groups and among unmarried or separated or divorced older people.

This study found that sociodemographic characteristics which are the rural residence and low education level were among the risk malnutrition among the elderly. Meanwhile, Chinese and Bumiputra Sarawak in ethnicity were found to be protective. A study conducted in Ethiopia found that the elderly in the rural area were about two times more likely to be malnourished [26]. Another study in Northern Italy found that the elderly with low education level were about three times more likely to have a risk for malnutrition [27]. Education is an essential factor to improve health status because higher education achievement effectively develops habits, skills, resources, and abilities that enable people to achieve a better life [28]. In terms of ethnicity, a healthy lifestyle including culturally low fat and cholesterol diet might be the reason that causes the Chinese and Bumiputra Sarawak to have fewer risks to malnutrition, but this needs to be studied further [29].

Depressed older people were two times more likely to be at-risk or malnourished. A study conducted among the Japanese older people in Kyoto found that depression was strongly associated with MNA-SF score ≤11 [30]. Similarly, a study conducted in South Africa reported that depressed older people were significantly about three times more likely to be at risk or be malnourished than those not depressed [31]. Comorbidities, lack of appetite, loss of interest in self-care, apathy, and physical weakness are the interconnected causes of depression among the elderly [32].

Other than depression, the elderly with IADL dependency were also found to be associated with at-risk or malnutrition. A study conducted among elderly outpatients in the Netherlands reported that depression and being IADL dependency were independently associated with an increased odd of at-risk of malnutrition and malnutrition with an adjusted odds ratio of 2.6 and 2.8, respectively [33]. A study among the elderly in the community in India also reported that the elderly who depend on IADL status were about three times more likely to be at-risk or malnourished [34]. Malnutrition is associated with the decline in functional condition as a result of impaired muscle function, decreased bone mass, immune dysfunction, anaemia, reduced cognitive function, poor wound healing, and social aspects such as social isolation and loneliness [35].

Low quality of life is also a predictor of at-risk or malnutrition among the elderly in Malaysia. A previous study suggested that the elderly with malnutrition were more likely to experience poor quality of life [36]. Evidence from both cohort studies and intervention trials also showed that improvement of nutritional status could lead to signiﬁcant improvements in quality of life in both physical and mental components [37].

The elderly with body mass index (BMI) below 18.5 kgm^−2^ were about six times more likely to be at risk of malnutrition or malnourished in this study. BMI is often used as a crude screening tool for nutritional status in a primary health care setting. A survey among the elderly in Australia identified that the elderly who were classified as at-risk or malnourished had a significantly lower mean weight and BMI than those classified as well-nourished [38]. The same study also found all elderly with BMI less than 18.5 kgm^−2^ were at risk or malnutrition. Meanwhile, our study also found similar findings; the elderly with BMI more than 25 kgm^−2^ were protective against at-risk or malnutrition. However, BMI may be regarded as a tool for rapid insight for nutritional screening but should not be used to replace exact nutritional evaluation since malnutrition or malnutrition risk may be present even in higher BMIs [39].

Food insecurity was another predictor for at-risk or malnutrition among the elderly. The previous study of Turkish elderly people showed that those who were food insecurity were around two times more likely to be at-risk or malnutrition [40]. Food insecurity affects the elderly the most that the elderly with food insecure need more health services and social support. The food insecurity itself links to low income, low educational status, social isolation and living alone, minority status, functional impairments, and neighbourhood walkability [40, 41].

Our study also found that inadequate plain water intake among the elderly was a significant risk factor to at-risk or malnutrition. Evidence suggests that the elderly have a higher baseline for osmolality and thus a higher osmotic operating point for a sense of thirst with little or no change in sensitivity, and indicates dwindled thirst in response to hypovolemia and hypervolemia of baroreceptors. For this reason, the means of water intake might be decreased as age increased [42]. Drinking plain water and other fluids is fundamental to maintain optimum health and well-being. However, age-related changes cause the elderly to be more vulnerable to water imbalance, and many older adults do not reach their recommended daily intake of oral fluids [43].

Although the factors to at-risk or malnutrition have been identified, this study only recruited the elderly residing in the community only. The elderly in the institutionalized facilities were not included in this study. A current local survey found a higher prevalence of malnutrition (13%) and at-risk of malnutrition (37%) among the elderly in a care home [44]. The factors that are associated with at-risk or malnutrition in institutionalized elderly might be different compared with community-dwelling older people. Another limitation of this study is the inability to identify the association between other variables such as household income and also undiagnosed for the non-communicable diseases with at-risk and malnutrition because it is not collected or measured due to the cost and not fulfilling the requirement of this study. Even with some of those shortcomings, our study provides nationally representative data for other associated factors at-risk or malnutrition among community-dwelling older people in Malaysia. These data are believed to be hugely beneficial to the policymakers and program managers in the planning of better health services for the elderly in Malaysia.

## 5. Conclusions

In conclusion, rural areas, low education level, depression, IADL dependency, low quality of life, underweight, food insecurity, and low plain water intake were significant risk factors for at-risk of malnutrition or malnutrition. A healthy ageing nation can be achieved provided early intervention is done as most of the factors identified are modifiable factors. Therefore, more effectively, multiagency collaboration can be executed to enhance the welfare of the elderly in Malaysia.

## Figures and Tables

**Table 1:** Sociodemographic characteristics of the respondents (*N* = 3977).

Sociodemographic characteristic	Number of samples	Percentage (%)
*Sex*		
Men	1872	47.1
Women	2105	52.9

*Strata*		
Urban	1689	42.5
Rural	2288	57.5

*Age groups (years)*		
60–69	2563	64.4
70–79	1104	27.8
80 and above	310	7.8

*Ethnicity*		
Malay	2591	65.1
Chinese	710	17.9
Indian	126	3.1
Bumiputra Sabah	278	7.0
Bumiputra Sarawak	158	4.0
Others	114	2.9

*Marital status*		
Never married	87	2.2
Married	2623	66.0
Separated or divorced	64	1.6
Widow or widower	1200	30.2

*Education*		
No formal/primary education	2745	69.0
Secondary education	967	24.3
Tertiary education	265	6.7

*Working status*		
Employed	1050	26.4
Unemployed	2927	73.6

*Individual monthly income*		
<RM1000.00	2519	64.1
RM1000.00–RM1999.99	845	21.5
≥RM2000.00	567	14.4

**Table 2:** Prevalence of malnutrition and risk of malnutrition among the elderly in Malaysia (prevalence (95% CI)).

Variable	MNA-SF status			*p* value
	Malnutrition	Risk of malnutrition	Normal	
All	7.3 (6.0–8.9)	23.5 (21.2–26.0)	69.2 (66.272.0)	—
*Sex*				
Men	7.2 (5.9–8.8)	22.9 (20.1–26.0)	69.9 (66.6–73.1)	0.718
Women	7.4 (5.6–9.7)	24.1 (21.3–27.2)	68.4 (64.5–72.1)	

*Strata*				
Urban	6.3 (4.8–8.4)	21.1 (18.3–24.2)	72.6 (68.7–76.2)	0.001^*∗*^
Rural	10.0 (8.2–12.2)	30.2 (27.0–33.7)	59.8 (55.9–63.5)	

*Age groups (years)*				
60–69	3.9 (3.0–5.1)	20.5 (17.7–23.6)	75.6 (72.3–78.6)	0.001^*∗*^
70–79	11.4 (8.3–15.5)	27.8 (24.3–31.6)	60.8 (55.9–65.4)	
80 and above	23.3 (18.3–29.3)	35.3 (27.3–44.3)	41.3 (34.3–48.7)	

*Ethnicity*				
Malay	7.9 (6.2–10.1)	24.3 (21.5–27.4)	67.7 (64.1–71.2)	0.023^*∗*^
Chinese	4.7 (3.1–7.1)	19.4 (15.1–24.6)	75.9 (70.0–80.9)	
Indian	11.0 (6.0–19.2)	24.9 (15.4–37.4)	64.2 (50.8–75.6)	
Bumiputra Sabah	10.7 (6.7–16.9)	30.6 (23.5–38.7)	58.7 (48.4–68.3)	
Bumiputra Sarawak	5.6 (3.4–9.1)	25.7 (21.1–31.0)	68.7 (63.7–73.4)	
Others	9.1 (3.5–21.6)	34.7 (24.3–46.9)	56.1 (39.2–71.8)	

*Marital status*				
Never married	6.2 (2.6–13.8)	36.0 (20.6–54.9)	57.8 (41.0–72.9)	0.003^*∗*^
Married	5.5 (4.4–7.0)	20.6 (18.3–23.1)	73.8 (71.0–76.5)	
Separated or divorced	20.4 (3.8–62.3)	30.2 (15.9–49.7)	49.4 (27.9–71.1)	
Widow or widower	11.0 (8.9–13.6)	29.0 (25.8–32.3)	60.0 (56.5–63.4)	

*Education*				
No formal/primary education	10.3 (8.5–12.4)	28.4 (25.6–31.3)	61.4 (57.8–64.8)	0.001^*∗*^
Secondary education	3.1 (2.1–4.8)	17.5 (14.2–21.5)	79.3 (75.5–82.7)	
Tertiary education	3.5 (1.6–7.2)	14.4 (9.8–20.7)	82.1 (76.0–87.0)	

*Working status*				
Employed	3.8 (2.6–5.3)	23.7 (20.1–27.7)	72.5 (68.4–76.3)	0.001^*∗*^
Unemployed	8.5 (6.9–10.3)	23.5 (21.0–26.1)	68.1 (64.8–71.1)	

*Individual monthly income*				
<RM1000	9.1 (7.4–11.2)	26.1 (23.4–28.9)	64.8 (61.3–68.2)	0.001^*∗*^
RM1000–RM1999	6.4 (4.3–9.5)	22.7 (18.9–26.9)	70.9 (66.1–75.3)	
≥RM2000.00	2.4 (1.4–4.1)	16.6 (12.4–21.8)	81.0 (75.5–85.5)	

^*∗*^Significant <0.05 for Rao-Scott adjusted chi-square statistic.

**Table 3:** Associated factor to at-risk or malnutrition among the elderly in Malaysia.

Variable	Unadjusted odd ratio		Adjusted odd ratio	
	OR	95% CI	aOR	95% CI
*Sex*				
Men	1		1	
Women	1.073	0.898–1.283	0.820	0.620–1.083

*Strata*				
Urban	1		1	
Rural	1.784^*∗*^	1.394–2.283	1.429^*∗*^	1.073–1.903
*Age groups (years)*				
60–69	1		1	
70–79	1.999 ^*∗*^	1.632–2.447	1.082	0.846–1.382
80 and above	4.395 ^*∗*^	3.113–6.205	0.987	0.593–1.642

*Ethnicity*				
Malay	1		1	
Chinese	0.667^*∗*^	0.485–0.916	0.583^*∗*^	0.377–0.902
Indian	1.173	0.645–2.133	1.392	0.788–2.458
Bumiputra Sabah	1.477	0.938–2.327	1.043	0.640–1.700
Bumiputra Sarawak	0.956	0.726–1.259	0.478^*∗*^	0.307–0.744
Others	1.640	0.810–3.318	0.995	0.541–1.829

*Marital status*				
Married	1		1	
Never married separated/widow	1.940^*∗*^	1.661–2.267	1.204	0.895–1.620

*Education*				
No formal/primary	2.898	1.983–4.235	1.741^*∗*^	1.023–2.961
Secondary	1.199	0.772–1.861	1.172	0.727–1.890
Tertiary	1		1	

*Working status*				
Employed	1		1	
Unemployed	1.239^*∗*^	1.035–1.484	0.918	0.700–1.205

*Individual monthly income*				
<RM1000.00	2.318^*∗*^	1.696–3.170	0.973	0.660–1.435
RM1000.00–RM1999.99	1.756^*∗*^	1.213–2.542	1.283	0.867–1.898
≥RM2000.00	1		1	

*Depression*				
Yes	4.859^*∗*^	3.460–6.823	2.086^*∗*^	1.433–3.037
No	1		1	

*Hearing disability*				
Yes	4.382^*∗*^	2.8836.658	1.644	0.836–3.234
No	1		1	

*Vision disability*				
Yes	3.609^*∗*^	2.279–5.716	1.197	0.726–1.976
No	1		1	

*ADLs status*				
Dependent	4.298^*∗*^	3.294–5.608	1.350	0.990–1.840
Independent	1		1	

*IADLs status*				
Dependent	3.261^*∗*^	2.674–3.976	1.355^*∗*^	1.053–1.743
Independent	1		1	

*Fall history*				
Yes	1.444^*∗*^	1.117–1.867	1.130	0.807–1.583
No	1		1	

*Social support*				
Low to fair	2.522^*∗*^	1.9463.269	1.172	0.802–1.713
High	1.162	0.886–1.525	0.901	0.622–1.306
Very high	1		1	

*Quality of life status*				
Tertile 1 (<44)	4.527 ^*∗*^	3.250–6.308	1.879^*∗*^	1.334–2.646
Tertile 2 (45–51)	1.749 ^*∗*^	1.324–2.310	1.562^*∗*^	1.114–2.190
Tertile 3 (≥52)	1		1	

*BMI status*				
Underweight	7.551^*∗*^	4.682–12.180	6.725^*∗*^	4.071–11.110
Normal	1		1	1
Overweight	0.525^*∗*^	0.417–0.660	0.538^*∗*^	0.419–0.691
Obese	0.503^*∗*^	0.344–0.736	0.501^*∗*^	0.306–0.820

*Food security status*				
Food secure	1		1	
Food insecure	3.068^*∗*^	2.445–3.850	1.767^*∗*^	1.366–2.287

*Fruit intake*				
<2 servings	1.352	0.982–1.860	0.960	0.647–1.423
≥2 servings	1		1	

*Vegetable intake*				
<3 servings	1.045	0.724–1.509	0.946	0.633–1.414
≥3 servings	1		1	

*Plain water intake*				
<6 servings	2.863^*∗*^	2.230–3.676	1.352^*∗*^	1.010–1.809
6–8 servings	1.758^*∗*^	1.291–2.393	1.446^*∗*^	1.010–2.071
>8 servings	1		1	

*Diabetes status*				
Yes	1.056		1.121	0.870–1.281
No	1		1	0.851–1.475

*Hypertension status*				
Yes	0.983	0.826–1.169	1.188	0.984–1.434
No	1		1	

*Hypercholesterolemia*				
Yes	0.863	0.709–1.051	0.891	0.679–1.168
No	1		1	

^*∗*^Significant <0.05 for logistic regression.

## Data Availability

The primary data used to support the findings of this study are available from the corresponding author's institution (National Institutes of Health, Ministry of Health Malaysia) upon request.
